# Reproductive hormones and sex chromosomes drive sex differences in the sleep–wake cycle

**DOI:** 10.3389/fnins.2024.1478820

**Published:** 2024-10-31

**Authors:** Micah Ralston, J. Christopher Ehlen, Ketema Paul

**Affiliations:** ^1^Department of Integrative Biology and Physiology, College of Life Sciences, University of California, Los Angeles, Los Angeles, CA, United States; ^2^Department of Neurobiology, Morehouse School of Medicine, Atlanta, GA, United States

**Keywords:** sex chromosome, gender differences, estrogen, androgen, NREM, REM

## Abstract

There are well-documented gender differences in the risk and severity of sleep disorders and associated comorbidities. While fundamental sex differences in sleep regulatory mechanisms may contribute to gender disparities, biological responses to sleep loss and stress may underlie many of the risks for sleep disorders in women and men. Some of these sex differences appear to be dependent on sex chromosome complement (XX or XY) and the organizational effects of reproductive hormones. Reproductive development plays a critical role in the ability of sex chromosomes and reproductive hormones to produce sex differences in sleep and wakefulness. Rodent models reveal that reproductive hormones drive many but not all sex differences in sleep–wake architecture. The ability of reproductive hormones to alter sleep are often dependent on responses to sleep loss and stress. However, in the absence of reproductive hormones (in gonadectomized rodents) sex differences in sleep amount and the ability to recover from sleep loss persist. The suprachiasmatic nucleus (SCN) and the ventrolateral preoptic nucleus (VLPO) of the hypothalamus play crucial regulatory roles in mediating the effects of reproductive hormones on the sleep–wake cycle. Taken together, the work reviewed here reveals that the reproductive hormone environment and sex chromosome complement may underlie gender disparities in sleep patterns and the risk for sleep disorders.

## Introduction

Getting sufficient amounts of daily sleep is critical for the health and well-being of women and men. However, sleep cycles across our society exhibit a variety of gender differences which include daily sleep amount (Saelee et al., 2023), daytime sleepiness (Gonsalvez et al., 2022), and responses to sleep medications (Roehrs and Roth, 2016). In clinical and epidemiological studies, women report more difficulties sleeping, restlessness upon awakening, and sleepiness across the daytime compared to men (Armitage et al., 2000; Jung et al., 2013; Saelee et al., 2023). Moreover, there are well-documented gender differences in the risk and severity of sleep disorders such as insomnia (Zhang and Wing, 2006), hypersomnia (Deshaies-Rugama et al., 2024*)*, and delayed sleep phase disorder (Reis and Paiva, 2019*)*. The origins of these gender differences are complex but often arise from difficulties recovering from sleep loss and stress. While gender disparities in sleep impairments are moderate under normal environmental conditions, they are often amplified under challenging conditions such as daily insufficient sleep and chronic stress. Importantly, gender differences in sleep impairments contribute to gender disparities in the prevalence of comorbidities such as depression and anxiety disorders (Joshi et al., 2023). These gender differences likely contribute to health disparities in a variety of sleep disorders and comorbid disorders, which is why revealing potential mechanisms that may underlie them is so important.

Although influenced by environmental and psychosocial factors, gender disparities in sleep are largely driven by biological sex differences in sleep regulatory mechanisms. For the remainder of this review, we will refer to sex differences instead of gender differences since we will report findings from animal models. Reproductive processes underlie several sex differences in the sleep–wake cycle. They have regulatory influences on several indices of sleep quality, such as sleep duration and frequency of daytime naps (Alzueta and Baker, 2023; Maki et al., 2024). Sex differences in sleep phenotypes are prevalent across the animal kingdom. To this end, animal models of biological sex are useful tools for identifying the roles of sex characteristics and reproductive processes on the sleep–wake cycle.

## Sleep regulatory mechanisms exhibit fundamental sex differences

Sleep is regulated by circadian and homeostatic mechanisms, as described by the two-process model (Borbely, 1982). Process C is the circadian process in which an endogenous clock times the sleep–wake cycle and consolidates sleep to a particular time of day. In diurnal species like humans, most sleep occurs at night, while in nocturnal species like mice, most sleep occurs during the day. Process S, sleep homeostasis, is based on prior sleep and wake amount. Sleep loss is followed by a sleep rebound characterized by increased NREM and REM sleep amounts and increased NREM slow wave activity (SWA). Sex differences in the circadian regulation of sleep and sleep homeostasis have been documented in primates (Laska and Tutsch, 2000; Barger et al., 2010), rodents (Swift et al., 2020; Chiem et al., 2024), and invertebrates (Hendricks et al., 2003; Wu et al., 2018). In mice, females have more consolidated but less total sleep throughout the day (Paul et al., 2006; Wu et al., 2018). Male mice are able to recover from sleep loss more quickly than females (Paul et al., 2006). Homeostatic responses to sleep loss also exhibit sex differences in gene expression. Transcripts for brain-derived neurotropic factor (BDNF), FosB, and the adenylate cyclase Adcy7 exhibit robust sex differences in brain expression after sleep deprivation (Shi et al., 2024). These findings show that animal models have promise to help reveal the mechanisms responsible for sex differences in the sleep–wake cycle.

Mouse models of human neurological disorders also exhibit sex differences in the sleep–wake cycle. For instance, in a mouse model of Huntington’s disease, BACHD, males recapitulate sleep phenotypes that are common in Huntington’s patients, but females do not (Chiem et al., 2024). A mouse model of Alzheimer’s disease reveals that female 5xFAD mice exhibit more cognitive benefits than males when exposed to feeding times that improve sleep efficiency (Campbell et al., 2024). Also, the Shank3 mouse model of autism spectrum disorder reveals a remarkable sex difference in which early-life sleep disruption improves general sociability in males while reducing risk aversion in females (Lord et al., 2022). Studies like these exemplify the value of mouse models of human disorders to understand the importance of sex differences in sleep in the risk, severity, and treatment of neurological disorders.

## Sex chromosomes contribute to sex differences in sleep

Sex chromosome complement (XX or XY) and the Y-linked *Sry* (sex-determining region Y) gene have regulatory influences on sleep (Ehlen et al., 2013). Although some sex differences in spontaneous sleep amount are dependent on reproductive hormones, other sex differences in sleep amount during the active phase are preserved after gonadectomy (GDX) and may be driven by non-hormonal factors. The four-core genotype (FCG) mouse model offers a robust framework for investigating the persistent sex differences following GDX. This model was generated by removing the *Sry* gene from the Y chromosome and reinserting it onto an autosome, resulting in progeny with discordant phenotypes and genotypes—of specific interest are XX mice with testes (XXM), and XY mice with ovaries (XYF). Mice from the FCG mouse model exhibit no sex differences in rest-phase sleep amount; however, during the active-phase (nighttime), XX males have more spontaneous NREM sleep than XX females. The XY male and female mice do not exhibit sex differences in sleep amount. During rebound from forced wakefulness, there is a change in the factors regulating sleep. XY females sleep more during their mid-active phase than XX females and have higher NREM SWA (Ehlen et al., 2013). These findings suggest that processes that regulate sleep homeostasis are sex-linked, and that sleep amount and sleep propensity are regulated differently in males and females during rebound from sleep loss.

It is unclear if the effects of *Sry* or sex chromosomes are due to the constitutive expression of sex-linked genes or the organizational influences of reproductive hormones during neonatal or pubertal development. Reproductive hormones have two primary effects on brain structures and secondary sex characteristics: organizational and activational (Arnold and Gorski, 1984). In most mammals, the reproductive hormone environment around the time of birth plays a major role in “organizing” neural circuitry (Han and De Vries, 2003; Morris et al., 2004). The activation of sex-related behaviors by the reproductive hormone environment in adulthood depends on the organizational effects of reproductive hormones during the perinatal environment and pubertal development. The synthesis and secretion of androgens and estrogens in mammals has a variable longitudinal profile during neonatal and juvenile development. Though the profile of reproductive hormone secretion exhibits several prolonged periods of stability, abrupt and dramatic adjustments in the magnitude and timing of their secretions are hallmarks of ontogeny.

In the FCG mouse line, mice that express the *Sry* gene exhibit more NREM sleep and total sleep during the active phase than mice that do not express *Sry*. *Sry* drives active phase “siesta” sleep amount on both XX and XY chromosomal backgrounds (Ehlen et al., 2013). However, all mice that express *Sry* develop into phenotypic males, regardless of the sex chromosome complement. This is the result of the organizational effect of androgens during perinatal development and the activational effects of androgens during pubertal development and adulthood. Early work has shown that the organizational effects of reproductive hormones in neonatal male and female rats are responsible for some of the activational effects of reproductive hormones on sleep in adulthood (Branchey et al., 1971). A seminal animal study reported that neonatal castration alters sleep responses to estradiol and progesterone injections in adult male rats (Branchey et al., 1973). In this study, perinatally feminized (castrated) male mice exhibited decreased REM and NREM sleep amounts in response to exogenous female reproductive hormones. These data demonstrate that reproductive hormones have organizational influences on sleep–wake architecture.

In adult GDX FCG mice, sex differences in spontaneous sleep phenotypes are coincident with the presence or absence of *Sry.* This association with *Sry* may be attributable to the activity of reproductive hormones prior to GDX. Following sleep deprivation, sex differences in recovery sleep in GDX wild-type mice are well established (Paul et al., 2006; Paul et al., 2009a) and differences between XX female and XY male mice recapitulate this male–female difference. In both spontaneous and recovery sleep, there is a positive effect of *Sry* on NREM, REM and total sleep in XX mice. However, in XY mice, the positive effect of *Sry* appears to be specific to spontaneous sleep but not recovery from sleep loss. These findings reveal that the association of *Sry* with sleep state is dependent on the underlying sex chromosome complement. More importantly, the specific effect of sex chromosomes appears to be restricted to homeostatic regulation of NREM sleep. The positive effect of *Sry* on REM sleep is conserved between baseline and sleep deprivation conditions; however, sleep deprivation reveals an interaction of *Sry* with sex chromosomes during REM sleep, with *Sry* having a positive effect on REM in XX mice, and no effect within XY mice (Ehlen et al., 2013). Thus, increasing homeostatic sleep pressure changes the relationship between sex chromosome complement and the *Sry* gene. These findings provide evidence that sex chromosomes, and sex-linkage, may play regulatory roles on the ability to recover from sleep loss.

## Reproductive hormones contribute to sex differences in sleep

Reproductive hormones have constitutive effects on sleep–wake architecture, sleep fragmentation, and sleep homeostasis (the ability to recover from sleep loss). In rats, the hormonal estrous cycle produces fluctuations in sleep–wake amount that include decreases in sleep amount during proestrus and a concomitant increase in wakefulness (Swift et al., 2020). NREM slow wave activity (SWA), a measurement of sleep pressure was also enhanced during proestrus. Sex differences in total sleep amount and sleep–wake fragmentation during spontaneous (sleep-replete) states are largely driven by 17β-estradiol and testosterone (Paul et al., 2006; Paul et al., 2009b; Schwartz and Mong, 2013; Cusmano et al., 2014; Choi et al., 2021). GDX in male and female mice and rats eliminates many sex differences in sleep–wake architecture (Paul et al., 2006; Cusmano et al., 2014). However, several sex differences in the homeostatic mechanism that regulates the ability to recover from sleep loss persist following GDX.

Sex differences in the ability of stress to increase REM sleep amount are also largely driven by reproductive hormones (Jefferson et al., 2014). These findings suggest that sex differences in responses to sleep loss differ from sex differences in responses to stress modalities that activate physiological stress response mechanisms. Stated differently, homeostatic sleep responses to stress are modulated by the reproductive hormone environment, whereas the effects of sleep loss remain largely unaffected by these hormonal influences. Sex differences in sleep responses to stress are partially encoded by nitric oxide synthase pathways in the basal forebrain (Kalinchuk et al., 2010; Chiem et al., 2021). The combined results from these studies demonstrate (1) the regulatory roles of sex chromosomes and reproductive hormones on sleep regulatory mechanisms, (2) that sex differences in sleep responses to stress are driven by different mechanisms than those that drive sleep homeostasis, and (3) that sex-linked factors constitutively drive sex differences in homeostatic sleep responses.

In rats, reproductive hormones organize neonatal sleep regulatory pathways in a way that primes adult sleep responsivity to the reproductive hormone environment (Cusmano et al., 2014). This study revealed that neonatal exposure to androgens reorganizes the sleep regulatory pathways of female rats. This work also revealed that most of the influences of androgens on sleep are driven by the aromatization of testosterone into estradiol. Work in this area could be pivotal in understanding the etiology of sex differences in the prevalence of sleep disorders and could provide important clues about the potential for sleep impairments to be co-morbid in other diseases and disorders that exhibit gender disparities.

## Brain sleep-regulatory mechanisms

Several systems that regulate circadian rhythms and sleep homeostasis have sensitivity to the reproductive hormone environment. The suprachiasmatic nucleus (SCN), located in the anterior hypothalamus, is a primary circadian regulatory region of the brain. The SCN is responsible for the daily timing and coordination of sleep and wake onsets and the diurnal consolidation of sleep and wake states across the 24-h day (Ono et al., 2024). The SCN exhibits several sexual dimorphisms, many of which are sensitive to the reproductive environment. For instance, female mice have a larger number of estrogen receptor (ER)-expressing neurons in the SCN than male mice (Vida et al., 2008; Zuloaga et al., 2014), but male mice have a larger number of androgen receptor (AR)-expressing neurons in the SCN than females (Iwahana et al., 2008). These dimorphisms largely drive sex differences in circadian locomotor activity (Vida et al., 2008). The VLPO is an important component of the system responsible for sleep homeostasis (Alam et al., 2014). VLPO neurons are responsible for inhibiting arousal-promoting centers of the brainstem and hypothalamus (Saper et al., 2005). The VLPO has been revealed to be a critical region for the effects of reproductive hormones on sleep. Neurons in the VLPO exhibit sexual dimorphisms that are sensitive to the reproductive hormone environment (Peterfi et al., 2004). The wake-promoting effects of estradiol in mice are driven by inhibition of sleep-active neurons in the VLPO (Deurveilher et al., 2008). The ability of estradiol to suppress REM sleep is related to the inhibitory effects of estrogen receptor activation in the VLPO (Hadjimarkou et al., 2008). In these studies, animal models have revealed potential brain-mediated mechanisms through which reproductive processes contribute to sex differences in the sleep–wake cycle. Still, more work needs to be done to determine the degree to which they underlie health disparities in sleep disorders.

## Conclusion

While the origins of gender disparities in the risk and severity of sleep disorders are elusive, sleep research in model systems reveals that sex chromosome complement and the reproductive hormone environment play critical roles in the organization of the sleep–wake cycle. Gonadectomies in male and female mice reduce many sex differences in sleep architecture, yet many sex differences in the ability to recover from sleep loss remain. Moreover, the ability of reproductive hormones to influence sleep is largely determined by early exposure. The studies described in this review reveal that while the hormone environment causes some sex differences in sleep, others are ingrained at a more fundamental, perhaps genetic, level. By gaining a greater understanding of the systems that underlie these sex differences, we are likely to improve our ability to treat sleep disorders and associated co-morbidities.

Understanding the mechanism of interaction between sex chromosome complement and reproductive hormones is crucial, not only for advancing knowledge in this field but also for the potential impact on public health. Due to a predominant focus on male subjects in animal research, this area has been historically underexplored. The neglect of sex as a biological variable in past research has led to a significant knowledge gap regarding such mechanisms in females, and thus the contribution to observed differences in sleep regulation and related disorders is unclear. The notion that certain sex differences in sleep may be “hard-wired” underscores the importance of early developmental processes, including the differentiation of sex chromosomes and the organizational effects of reproductive hormones. Future research should aim to dissect the relative contributions of sex chromosomes versus reproductive hormones in shaping these differences. Ongoing studies investigating rodent models throughout development, along with explorations of stress and sleep loss under varying hormonal environments, could reveal new pathways that are susceptible to intervention. These inquiries will likely yield critical insights into the etiology of sex-specific sleep disorders and allow us to move toward more inclusive and effective health strategies.
